# Implementing a Digital Handover System to Improve Safety and Efficacy of Handover Across Acute Psychiatric Inpatient Sites

**DOI:** 10.1192/bjo.2024.379

**Published:** 2024-08-01

**Authors:** Aaron Harris

**Affiliations:** Oxleas NHS Foundation Trust, London, United Kingdom

## Abstract

**Aims:**

To implement a digital handover system within Oxleas inpatient sites to improve the visibility of tasks both completed and pending, to reduce the number of tasks missed and to provide a clear audit trail relating to tasks handed over.

**Methods:**

Junior doctors providing on-call cover to acute sites across all 3 boroughs served by Oxleas were invited to complete a questionnaire relating to the efficacy of handover. With this data & information gathered through discussions with the trust's informatics team, a digital handover system, based in Microsoft Teams, was developed. This was piloted and refined through 6 PDSA cycles from September 2022 – August 2023 before being implemented across all Oxleas acute sites from August 2023. Further questionnaires were completed 1 month & 6 months after its roll out to assess the impact of the change.

**Results:**

Doctors were asked to complete a questionnaire at 3 time points: pre-intervention (T0, 20 respondents), 1-month post-intervention (T1, 13 respondents), and 6-months post-intervention (T2, 12 respondents).

- At T0, 92.3% of respondents reported tasks created by the on-call team had been missed due to staff not being aware, this reduced to 11.1% at T1, and 28.6% at T2.
- At T0, 23.1% of respondents agree/strongly agree that it is easy to view tasks that have been done on their ward out-of-hours.
- By comparison, at T1 69.2% reported the digital handover system has made it easier to view what had been done on a ward out-of-hours, rising to 83.3% at T2.
- At T1, 76.9% reported the digital handover system has made it easier to view tasks when on-call, rising to 83.3% at T2.
- At T0, 30% agree/strongly agree that the outgoing on-call doctor leaves a written record of tasks completed and outstanding. This rose to 69.3% at T1, and 41.7% at T2.

**Conclusion:**

There is strong evidence that effective handover is a key aspect of clinical care, and failure of this is a preventable cause of patient harm. The initial questionnaire highlighted issues with the efficacy and safety of the handover process within acute sites at Oxleas, which the digital system sought to address. After implementation of the digital system, the findings demonstrated improvements in the handover process, with visibility increasing for tasks both completed & in progress, and fewer reports of tasks being missed by the ward-based doctors, which was maintained over the 6-month follow up period.

